# Pulmonary Involvement in Children With Systemic Lupus Erythematosus

**DOI:** 10.3389/fped.2020.617137

**Published:** 2021-02-02

**Authors:** Ge Dai, Linlin Li, Ting Wang, Wujun Jiang, Jie Ma, Yongdong Yan, Zhengrong Chen

**Affiliations:** ^1^Children's Hospital of Soochow University, Suzhou, China; ^2^Children's Hospital of Nanjing Medical University, Nanjing, China; ^3^Children's Hospital of Wujiang District, Suzhou, China

**Keywords:** systemic lupus erythematosus, pulmonary involvement, children, HRCT findings, anti-RNP, ANCA

## Abstract

**Background:** Symptomatic pulmonary involvement in systemic lupus erythematosus (SLE) seems not uncommon in children. However, there are few data on the characteristics and laboratory parameters of SLE patients with pulmonary involvement.

**Methods:** This was a hospital-based study involving 111 SLE patients from 1 January 2012 to 31 December 2016. The demographic, clinical, and laboratory data of the patients were prospectively collected. They were followed as outpatients until December 2019. Clinical characteristics and laboratory parameters of patients with and without pulmonary involvement were compared.

**Results:** Of the 111 patients with SLE, we identified 18 patients (16.2%) with pulmonary involvement. The most common HRCT findings were ground glass opacity, interlobular septal thickening, bilateral diffuse infiltrates, and pleurisy/pleural effusion (55.6, 50, 50, and 44.4%, respectively). SLE patients with pulmonary involvement tended to have a longer disease duration (14 [12–24.5] vs. 5 [2–9] months, *P* < 0.01). We also observed a significant association between the presence of anti-Sm antibody, ANCA, Anti-RNP and the presence of pulmonary involvement of SLE (all *P* < 0.001).

**Conclusions:** Lung involvement was frequent in SLE patients from Southeast China. Patients with a longer duration of symptoms before SLE diagnosis tended to have pulmonary involvement. When children with SLE are found to have anti-RNP antibody and positive ANCA, it should be alert to the occurrence of pulmonary involvement.

## Introduction

Systemic lupus erythematosus (SLE) is a complex autoimmune disorder of unknown etiology in which tissues and cells are damaged by pathogenic autoantibodies and immune complexes. Adult SLE and childhood SLE have similar clinical features, but children are known to have a more severe disease course. SLE is the most common connective tissue disease affecting the lung, with a similar proportion between adults and children. In children with SLE, it has been reported that pulmonary involvement occurred in 7.6–75% of the patients (1–3). The types of pulmonary manifestations reported involve any portion of the pulmonary organ system including the pleura, diaphragm, parenchyma, and vasculature.

Due to the potentially high prevalence of lung complications in SLE, assessing the risk factors that predict pulmonary manifestations is of great importance. It was reported that SLE duration, low complement levels, high anti-dsDNA levels and disease activity were the risk factor associated with the development of pulmonary manifestations in adult patients (4, 5). Nevertheless, there are few data on the characteristics and laboratory parameters of SLE patients with pulmonary involvement.

Therefore, in this study, we prospectively investigated the characteristics and laboratory parameters of SLE patients with pulmonary involvement.

## Method

### Patients and Study Design

This was a hospital-based study conducted at Children's Hospital of Soochow University located in the Southeast region of China. Patients diagnosed with SLE were prospectively included in our study from 1 January 2012 to 31 December 2016. They were followed consecutively as outpatients until December 2019. We enrolled patients aged <18 years who were diagnosed with SLE under the systemic lupus international collaborating clinics classification criteria for a diagnosis of SLE (6). Patients with mixed connective tissue diseases were excluded from the study. This investigation was conducted in accordance with the Declaration of Helsinki and was approved by the Ethics Research Committee of Children's Hospital of Soochow University.

### Data Collection

The demographic and clinical data of patients with SLE including patients' age at onset, gender, duration of symptoms before SLE diagnosis, follow-up duration, different clinical features at presentation. Laboratory data collected included hematological and immunological parameters consisting of the lowest levels of complement components 3 and 4 (C3 and C4) (low C3: <90 mg/dl; low C4: <20 mg/dl), anti-double stranded (ds) DNA antibody, anti-Smith (Sm) antibody, anti-neutrophil cytoplasmic antibodies (ANCA), anti-ribonucleoprotein (RNP) antibody, anti-SSA antibody and anti-SSB antibody. Renal biopsy data were also obtained.

Chest HRCT scans were performed when patients presented with persistent cough, dyspnea on exertion, shortness of breath, haemoptysis, or chest pain. Two senior radiologists independently reviewed HRCT scans, with each diagnosis reached by consensus. Furthermore, the presence of the following pulmonary HRCT manifestations was recorded as previously described (7, 8): pleurisy/pleural effusion, pneumonitis, interstitial lung disease, bronchiectasis, diffuse alveolar hemorrhage and pulmonary edema. Respiratory involvement was considered primary when it was directly related to SLE activity, and infections, drug toxicity, and neoplasia had been excluded.

Pulmonary function tests were performed when patients had abnormal HRCT findings. They were performed by spirometry (MasterScreen IOS; Jäger, Höchberg, Germany) and interpreted by a pediatric pulmonologist. The parameters measured were the forced expiratory flow volume in 1 second (FEV1), forced vital capacity (FVC) and FEV1/FVC. The patients performed three times of forced expiratory maneuvers from the total lung capacity to the residual volume, and the best of the three measurements was used in the final analysis.

### Statistical Analysis

We used n (%) for categorical variables and median (quartiles) for continuous variables with non-normal distribution or mean and standard deviation (SD) for those with normal distribution. We assessed differences in categorical variables with the χ^2^ test or Fisher exact test. We calculated 95 % confidence interval (95 % CI) for differences in medians with an exact test. SPSS (version 22.0) software was used for all statistical analysis.

## Results

A total of 111 SLE patients were included in our study. Of these, 73.0% were female and 27.0% were male; median age at SLE diagnosis was 11.3 (range 9.0–13.0) years. Median period of follow-up was 5.5 (range, 3.0–7.0) years. The presenting symptoms at diagnosis are summarized in Table 1. Of the 111 SLE patients, 73 (65.8%) patients received a renal biopsy. Cutaneous lesions (71.2%, *n* = 79), fever (56.8%, *n* = 63), nephritis (56.8%, *n* = 63) and hematological involvement (51.4%, *n* = 57) were the major clinical manifestations. Of the 63 patients with lupus nephritis, 26 (41.3%) had positive ANCA. IgA, IgG, IgM, C3, and C1q deposits by immunofluorescence were simultaneous detected in all the 26 ANCA positive patients, indicating lupus nephritis instead of ANCA associated vasculitis. Pulmonary involvement was found in 18 (16.2%) patients, yielding an overall prevalence of 16.2% (95% CI: 9.3–23.2). Among the 18 patients with pulmonary involvement, 15 patients had pulmonary involvement at diagnosis, 3 patients had pulmonary involvement during the treatment. All patients received prednisolone. Other immunosuppressive drugs given were mycophenolate mofetil (four patients), Hydroxychloroquine (two patients) and cyclophosphamide (two patients). No respiratory sequalae were observed in any patients during follow-up.

**Table 1:** Demographic and clinical characteristics at diagnosis of systemic lupus erythematosus (SLE) patients in this study (*n* = 111).

	**Patients *n* (%)**
**DEMOGRAPHIC CHARACTERISTICS**	
Female/male	1:0.37
Age at SLE diagnosis, median (range), y	11.3 (9.0–13.0)
**CLINICAL CHARACTERISTICS**, ***n*** **(%)**	
Fever	63 (56.8)
Cutaneous	79 (71.2)
Arthritis	22 (19.8)
Mucosal lesion	20 (18.0)
Nephritis	63 (56.8)
Pericarditis	18 (16.2)
Gastrointestinal involvement, except hepatitis	14 (12.6)
Hepatitis	14 (12.6)
Hematologic abnormalities	57 (51.4)
Neuropsychiatric manifestations	11 (9.9)
Pulmonary involvement	15 (13.5)

**Figure 1:**
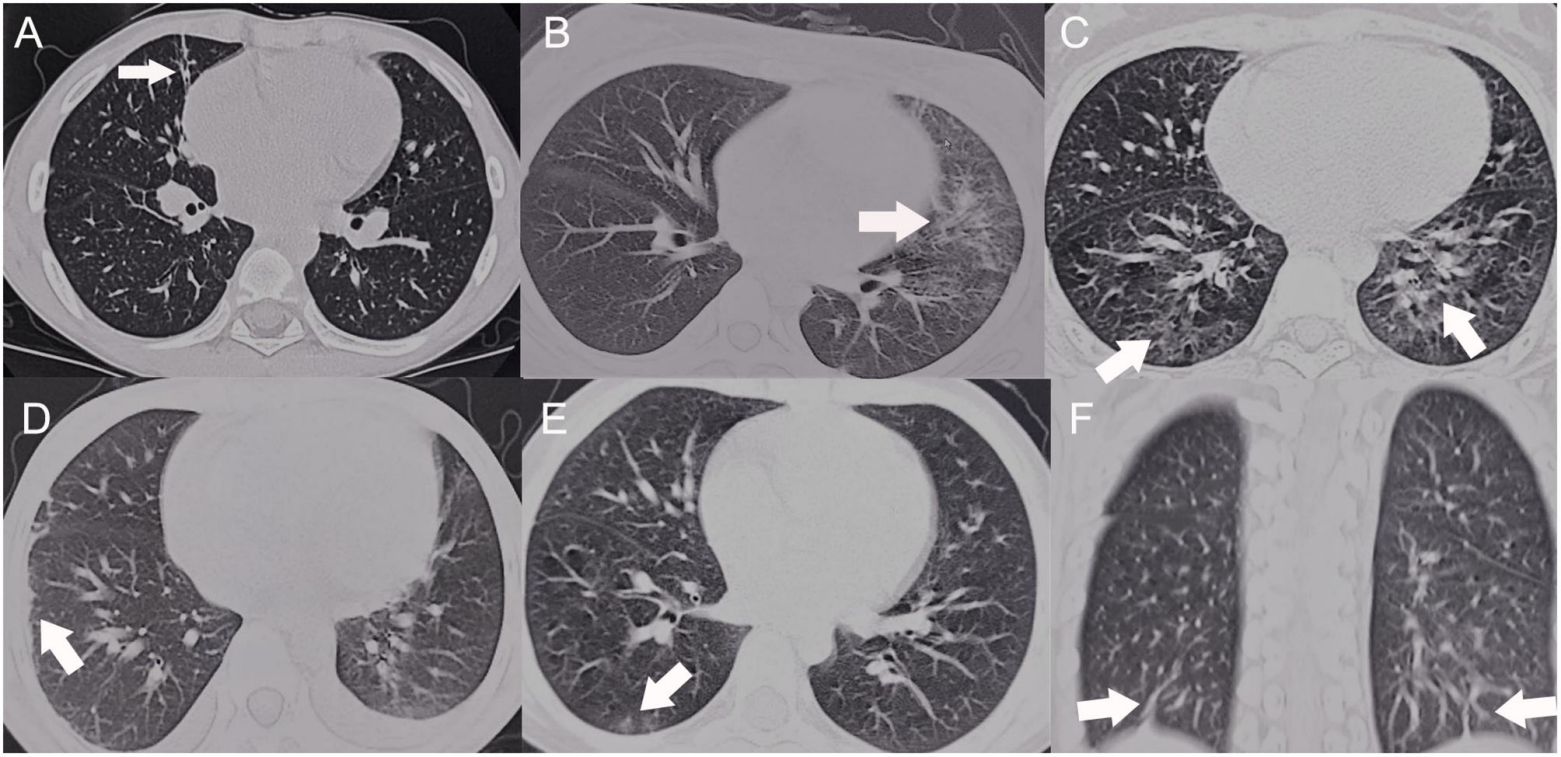
HRCT scans of the SLE patients with pulmonary involvement. **(A)** Fibrotic streak; **(B)** Reticular pattern; **(C)** Mosaic perfusion; **(D)** Pleural thickening **(E)** Ground glass opacity; **(F)** Subpleural interlobular septal thickening.

Data concerning HRCT scans of the SLE patients with pulmonary involvement are presented in Table 2. Ground glass opacity was found in 10 (55.6%) patients, interlobular septal thickening in 9 (50.0%) patients, bilateral diffuse infiltrates in 9 (50.0%) patients, pleurisy/pleural effusion in 8 (44.4%) patients, fibrotic streak in 6 (33.3%) patients and patchy infiltrates in 5 (27.8%) patients (Figure 1). Most of the abnormalities were distributed bilaterally (94.4%), in the lower lobes (82.3%) or subpleural regions (61.1%).

**Table 2:** Summary of HRCT findings in SLE patients showing pulmonary involvement.

**HRCT findings**	*N* (%) ^*^
**ABNORMALITIES**	
Ground glass opacity	10 (55.6)
Interlobular septal thickening	9 (50.0)
Bilateral diffuse infiltrates	9 (50.0)
Pleurisy/pleural effusion	8 (44.4)
Fibrotic streak	6 (33.3)
Patchy infiltrates	5 (27.8)
**DISTRIBUTION OF ABNORMALITIES**	
Bilateral	17 (94.4)
Lower lobes	14 (82.3)
Subpleural regions	11 (61.1)
Patchy random distributed	4 (22.2)

* *Multiple abnormalities were present in each patient*.

All the SLE patients with pulmonary involvement received pulmonary function tests. The median FVC (%predicted) and FEV1 (%predicted) were 64.8 (42.0–76.9) and 72.1 (51.9–82.9), respectively. The median value of FEV1/FVC was 115.5 (112.9–121.2). Abnormal pulmonary functions were found in 16 (88.9%) patients. All had reduced FVC or restrictive lung defects.

Table 3 described the demographic, clinical and laboratory data of SLE patients with and without pulmonary involvement. SLE patients with pulmonary involvement tended to have a longer duration of symptoms before SLE diagnosis (14 [12–24.5] vs. 5 [2–9] months, *P* < 0.01). SLE patients with pulmonary involvement were more likely to presented with pericarditis and neuropsychiatric manifestations (both *P* < 0.05). We also observed a significant association between the presence of anti-Sm antibody, ANCA, Anti-RNP and the presence of pulmonary involvement of SLE (all *P* < 0.001; Table 3).

**Table 3:** Demographic, clinical, and laboratory data of systemic lupus erythematosus (SLE) patients with and without pulmonary involvement (*n* = 111).

	**Pulmonary involvement (*n* = 18)**	**No pulmonary involvement (*n* = 93)**	***P***
**DEMOGRAPHIC CHARACTERISTICS**			
Female/male	1:0.29	1:0.39	0.77
Age at SLE diagnosis, median (quartile), y	11.4 (10.1–12.3)	11.3 (7.7–13.0)	0.75
Duration of symptoms before SLE diagnosis, median (quartile), m	14 (12–24.5)	5 (2–9)	<0.01
**CLINICAL CHARACTERISTICS**, ***n*** **(%)**			
Fever	11 (61.1)	52 (55.9)	0.80
Cutaneous	13 (72.2)	66 (71.0)	0.98
Arthritis	6 (33.3)	16 (17.2)	0.19
Mucosal lesion	5 (27.8)	15 (16.1)	0.31
Nephritis	14 (77.8)	49 (52.7)	0.07
Pericarditis	10 (55.6)	8 (8.6)	<0.001
Gastrointestinal involvement, except hepatitis	3 (16.7)	11 (11.8)	0.70
Hepatitis	4 (22.2)	10 (10.8)	0.24
Hematologic abnormalities	12 (66.7)	45 (48.4)	0.20
Neuropsychiatric manifestations	5 (27.8)	6 (6.5)	0.02
**LABORATORY FINDINGS**			
Low complement 3 level	16 (88.9)	58 (62.4)	0.03
Low complement 4 level	16 (88.9)	66 (71.0)	0.15
Anti-dsDNA antibody positivity	16(88.9)	82 (88.2)	1.00
Anti-Sm antibody positivity	11 (61.1)	14 (15.1)	<0.001
Anti-Ro antibody positivity	7 (38.9)	41 (44.1)	0.80
ANCA positivity	16 (88.9)	17 (18.3)	<0.001
Anti-RNP antibody positivity	12 (66.7)	15 (16.1)	<0.001
Anti-SSB antibody positivity	5 (27.8)	18 (19.4)	0.53

## Discussion

In the present study, we have determined the prevalence of pulmonary involvement in SLE to be 16.2% in the Southeast region of China. The frequency of symptomatic pulmonary involvement at diagnosis in children with SLE ranges from 7.6 to 75% (1, 9, 10). The wide range of prevalence found in the previous studies may be due to known racial and ethnic phenotypic variability, as well as different approaches taken to determine the presence of pulmonary involvement with SLE.

The types of pulmonary manifestations reported are diverse, and may involve any portion of the pulmonary organ system including the pleura, diaphragm, parenchyma, and vasculature (3). However, the most common pulmonary involvement appears to be pleuritis, which affects 12.5–32% of children with SLE during the course of their disease (2, 11). In our study, pleuritis affects 7.2% (8/111) of children with SLE. Moreover, 94.4% of the children with pulmonary involvement display bilateral presentation. The high prevalence of bilateral involvement in our study is in line with the previous studies (1, 9, 12).

The presence of anti-RNP antibody was described to be specific (specificity ranging from 84 to 100%) of mixed connective tissue disease (13). Previous reports found that positive anti-RNP antibody are risk factors for pulmonary arterial hypertension in patients with SLE (14–16). Anti-RNP positivity is also reported to be associated with a more frequent pulmonary involvement in other connective tissue disease (17). Our results are the first study that emphasize the role of anti-RNP positivity in predicting occurrence of pulmonary involvement in childhood SLE.

In our study, ANCA positivity was more commonly found in children with pulmonary involvement. ANCA is a group of autoantibodies against specific antigens such as neutrophil cytoplasmic granules and monocyte lysosomes. The discovery of ANCA helped establish ANCA-associated vasculitis as a separate and well-defined clinical entity (18, 19). Previous studies reported that the positive rate of ANCA in patients with SLE ranges from 25 to 69% (20–22). In our study, 33 of the 111 SLE children were positive for ANCA, with a positive rate of 29.7%, which was consistent with the previous reports. The relationship between positive ANCA and pulmonary involvement has also reported in previous studies, especially in adult patients (20, 22). Therefore, when the children with SLE is found to have positive ANCA, it should be alert to the occurrence of pulmonary involvement.

Our study has some limitations. First, the data which were collected from a single center may have introduced bias. Second, the total number included in our study is relatively small. Thus, a multicenter, prospective study is warranted to evaluate pulmonary manifestations in children with SLE.

## Conclusion

In conclusion, lung involvement was frequent in SLE patients from Southeast China. Patients with a longer duration of symptoms before SLE diagnosis tended to have pulmonary involvement. When children with SLE are found to have anti-RNP antibody and positive ANCA, it should be alert to the occurrence of pulmonary involvement.

## Data Availability Statement

The datasets generated for this study are available on request to the corresponding author.

## Ethics Statement

The studies involving human participants were reviewed and approved by Ethics Research Committee of Children's Hospital of Soochow University. Written informed consent to participate in this study was provided by the participants' legal guardian/next of kin.

## Author Contributions

WJ, TW, and JM were involved in designing the research, analyzing the data, and writing the manuscript. WJ, GD, and LL were also involved in supervising subjects' recruitment, data collection, and drafting the manuscript. YY and ZC had primary responsibility for the final content. All authors approved the final version of the manuscript.

## Conflict of Interest

The authors declare that the research was conducted in the absence of any commercial or financial relationships that could be construed as a potential conflict of interest.
